# Supplementary material for: Contrasting carbon cycle along tropical forest aridity gradients in West Africa and Amazonia
Source: Nat Commun. 2024 Apr 11;15:3158. doi: 10.1038/s41467-024-47202-x (PMC11009382; doi:10.1038/s41467-024-47202-x)
Supplement: Supplementary file 1 — Supplementary Information [file 41467_2024_47202_MOESM1_ESM.pdf]

Supplementary for

Contrasting carbon cycle along tropical forest aridity gradients in West Africa and Amazonia

Huanyuan **Zhang-Zheng** 1, 2 *, Stephen **Adu-Bredu** 3, 4, Akwasi **Duah-Gyamfi** 3, Sam **Moore** 1, Shalom D. **Addo-Danso** 3, Lucy **Amissah** 3, Riccardo **Valentini** 5, Gloria **Djagbletey** 3, Kelvin **Anim-Adjei** 3, John **Quansah** 3, Bernice **Sarpong** 3, Kennedy **Owusu-Afriyie** 3, Agne **Gvozdevaite** 1, Minxue **Tang** 6, Maria C. **Ruiz-Jaen** 7, Forzia **Ibrahim** 8, Cécile A.J. **Girardin** 1, Sami **Rifai** 9, Cecilia A. L. **Dahlsjö 1**, Terhi **Riutta** 1, Xiongjie **Deng** 1, Yuheng **Sun** 10, Iain Colin **Prentice** 6,11, Imma **Oliveras Menor** 1,12, Yadvinder **Malhi** 1, 2 *

*Corresponding Author

1 Environmental Change Institute, School of Geography and the Environment, University of Oxford, Oxford, United Kingdom,
2 Leverhulme Centre for Nature Recovery, University of Oxford, United Kingdom,
3 Forestry Research Institute of Ghana, Council for Scientific and Industrial Research, Kumasi, Ghana
4 Department of Natural Resources Management, CSIR College of Science and Technology, Kumasi, Ghana
5 Centro Euro-Mediterraneo sui Cambiamenti Climatici, Leece, Italy
6 Georgina Mace Centre for the Living Planet, Department of Life Sciences, Imperial College London, Silwood Park Campus, Buckhurst Road, Ascot, United Kingdom
7 Forestry Division, Food and Agriculture Organization of the United Nations, Panama City, Panama
8 Department of Forest Ecology, Faculty of Forestry and Wood Sciences, Czech University of Life Sciences, Praha, Czech Republic.
9 School of Biological Sciences, University of Adelaide, Adelaide, South Australia, Australia
10 Groningen Institute for Evolutionary Life Sciences, University of Groningen, P.O. Box 11103, 9700 CC Groningen, The Netherlands
11 Department of Earth System Science, Ministry of Education Key Laboratory for Earth System Modeling, Institute for Global Change Studies, Tsinghua University, Beijing, China
12 AMAP (Botanique et Modelisation de l'Architecture des Plantes et des Végétations), CIRAD, CNRS, INRA, IRD,Université de Montpellier, Montpellier, France

*Corresponding emails: huanyuan.zhang@ouce.ox.ac.uk; yadvinder.malhi@ouce.ox.ac.uk

Please refer to ‘figshare’ (https://doi.org/10.6084/m9.figshare.23615472) data and code deposits associated with this manuscript for the full data set of carbon budget. all_GPP_together_per_plot.csv contains measurements of each carbon cycle component as a mean per plot. all_GPP_together_per_SITE.csv contains measurements of each carbon cycle component as a mean per site.

Supplementary Method Field measurements and processing procedure

The procedure was written to ensure reproducibility of results and thus includes many processing details. Both Amazonia and West African aridity gradients show increasing seasonality toward dry sites. Since this paper focuses on the spatial variation of the carbon budget, not seasonal variation nor long-term spatial variation, here we average monthly measurements to an annual mean for both study gradients. Thus, the data processing procedure here may not be suitable for those focusing on seasonal variation.

Leaf area index (LAI) was estimated from hemispherical images taken with a Nikon 5100 camera and Nikon Fisheye Converter FC-E8 0.21x JAPAN near the center of each of the 25 subplots in each plot in each site, at a standard height of 1 m, and during overcast conditions. 22,000 photos were collected in total, every month during 2016-2017(ANK), 2012-2017 (BOB&KOG). Photos were processed using machine learning-based software 'ilastik' [1] for pixel classification and CANEYE [2] for leaf area index calculations. The exposure procedure followed [3] and GEM manual [4] (http://gem.tropicalforests.ox.ac.uk). The following parameters were supplied to CANEYE.

(1) P1 = angle of view of the fish eye divided by the amount of pixels from the centroid of the fish eye circle to where the horizon is on the image.
(2) angle of view = 90 degree, in which case, the edge of the photo is the horizon and the centroid of the image is zenith.

(3) COI = 80, consideration of field is 80 degrees, we don't want the edge of the photo because it is not clear and sometime obscure by tall grasses or saplings.

(4) Sub sample factor =1

(5) Fcover = 20 degree, this is to calculate the percentage of black pixels within the central 20-degree ring. We used this to understand the relative openness of canopy for the given image. It is not relevant to LAI

(6) PAIsat = 10, When a pixel is completely black, mathematically, the leaf area index (LAI) is infinite. As we provide CANEYE 25 subplot images for each estimation of LAI, this means all 25 subplot images show black at a given pixel. To address this 'infinite'issue, we use a value of 10 for LAI in such cases. This value is based on the guess that, the densest point in a tropical forest should have an LAI of 10.

(7) Latitude 0 and Day of Year a random number (not relevant for tropical site LAI)

Then, we extract output from CANEYE using software R. We chose the latest method of LAI calculation offered by CANEYE, 'CE V6.1 True PAI'. CANEYE reported one LAI value per method (4 methods) per plot per site per month, as a synthesis across 25 subplots images. As systematic error is dominating in LAI calculation, we take the standard deviation of LAI across four methods as the uncertainty for LAI.

Canopy respiration (R_leaf) is calculated as plot-mean LAI multiplied by plot-mean leaf dark respiration (Rdark), a leaf gas exchange measurement. To obtain the leaves, branches for both sun leaves and shade leaves were detached and immediately re-cut under water to restore hydraulic connectivity for subsequent gas exchange measurement. The leaves were fully darkened for 30 min prior to measuring Rdark. Rdark was measured using an open flow gas exchange system (LI-6400XT, Li-Cor Inc., Lincoln, NE, USA) and block temperature was kept constant throughout the sampling period at 30º C. The uncertainty of Rdark was calculated as the standard error of raw measurements [5]. We convert measurements of Rdark from 30 degree to mean annual air temperature following [6]. Rdark was measured for sun and shade leaves and from wet to dry seasons. We calculate a basal area community weighted mean for

Rdark_sun and Rdark_shade. Then, we calculate canopy respiration per plot using: R_leaf = Rdark_sun * F_sunlit + Rdark_shade* (1 – F_sunlit), where F_sunlit is the sunlit leaf area. It is calculated as Fsunlit = (1 – exp(− K∗LAI))/K where K is the light extinction coefficient [7]. The final canopy total respiration was calculated as R_leaf * 0.67 to account for daytime light inhibition of leaf dark respiration [8].

Above-ground live wood respiration (R_stem), was quantified at monthly intervals by measuring rates of CO2 accumulation to chambers attached to the tree trunk, and scaling using stem surface area allometries, using a previously-developed equation[9]. Bole respiration per unit surface area was measured using wood respiration closed dynamic chamber method, from at least 50 trees covering dominating species distributed evenly throughout each plot at 1.3 m height with an IRGA (EGM-4) and soil respiration chamber (SRC-1) connected to a permanent collar. The uncertainty of bole respiration per unit surface area was calculated as the standard error of raw measurements. To recognise the large uncertainty of total stem surface area, mostly due to the simple allometric equation, we assigned an uncertainty of 30%.

Coarse root respiration (R_coarse_root) was not measured, by estimated by R_stem multiplied by 0.21 ± 0.10, following [10–12].

Total soil CO2 efflux (R_soil), called R_soil_no_coarseroot_with_litter in the raw data sheet, was measured every month at the same point in each of the 25 sub-plots on each plot. It was measured using a closed dynamic chamber method with an infra-red gas analyser and soil respiration chamber (EGM-4 IRGA and SRC-1 chamber, PP Systems, Hitchin, UK) sealed to a permanent collar in the soil. Coarse root respiration was assumed missed by the above method

[12]. The uncertainty of R_soil_no_coarseroot_with_litter was calculated as the standard error of raw measurements.

Therefore, the R_soil_no_coarseroot_with_litter is composed of R_rhizosphere (including fine roots, mycorrhizal and exudates) respiration, soil organic matter derived respiration (R_soil_heterotrophic), and soil surface fine litter respiration (R_fine_litter). The percentage of each component was determined by using a partitioning experiment similar to that described in [13,14].

Root exudates NPP was not directly measured while mycorrhizal respiration was incorporated in R_rhizosphere in R_a, bringing uncertainty to GPP and CUE. Following [14], We estimated the root exudation rate from literature as (i) 6% of total NPP [15] (ii) 59% of root NPP [16], (iii) 37% of root respiration (calculated from data in [17])

Coarse woody debris respiration and dead wood respiration was not directly measured, which affect the estimates of carbon sink (net ecosystem exchange) but is irrelevant to GPP nor CUE. A study of Amazonia lowland intact forest found CWD respiration as 76% of CWD input, where a steady state (D_cwd = D_cwd_to_soil + R_cwd) was assumed [18]. However, the proportion of CWD respired could be rather variable [19]; A recent study at the Borneo lowland forests reported a 90% [20]. In this study, we estimated R_cwd as (0.9+0.76)/2 = 0.83 of D_cwd, with ±0.1 uncertainty.

This study sources stem biomass (or called above-ground coarse woody biomass, estimated from tree height and girth) and some NPP components from [21]. However, it is worth noting that the study is limited in that some minor components (in terms of magnitude) of the

carbon cycle were not covered by this study. For instance, Volatile organic compound NPP was found to be a very minor component of the carbon cycle of an Amazonian Forest [8]. Ground flora was neglected in ANK and BOB due to their relatively low abundance, and was included in KOG, a forest to savanna transition zone. Epiphytes and liana were also not counted albeit their wide existence in the field, especially in BOB01.

When combining or multiplying different components of the carbon cycle, uncertainties were propagated following [8].

Supplementary 2: Correlation between NPP and GPP

Here we presented the correlation between Gross primary productivity (GPP), net primary productivity (NPP) and carbon use efficiency (CUE). Please note that GPP and NPP in this study are calculated as the sum of various components (see Supplementary Data 1). CUE is calculated as NPP/GPP. The regression shows for example how well the spatial variation of GPP captured the spatial variation of NPP.

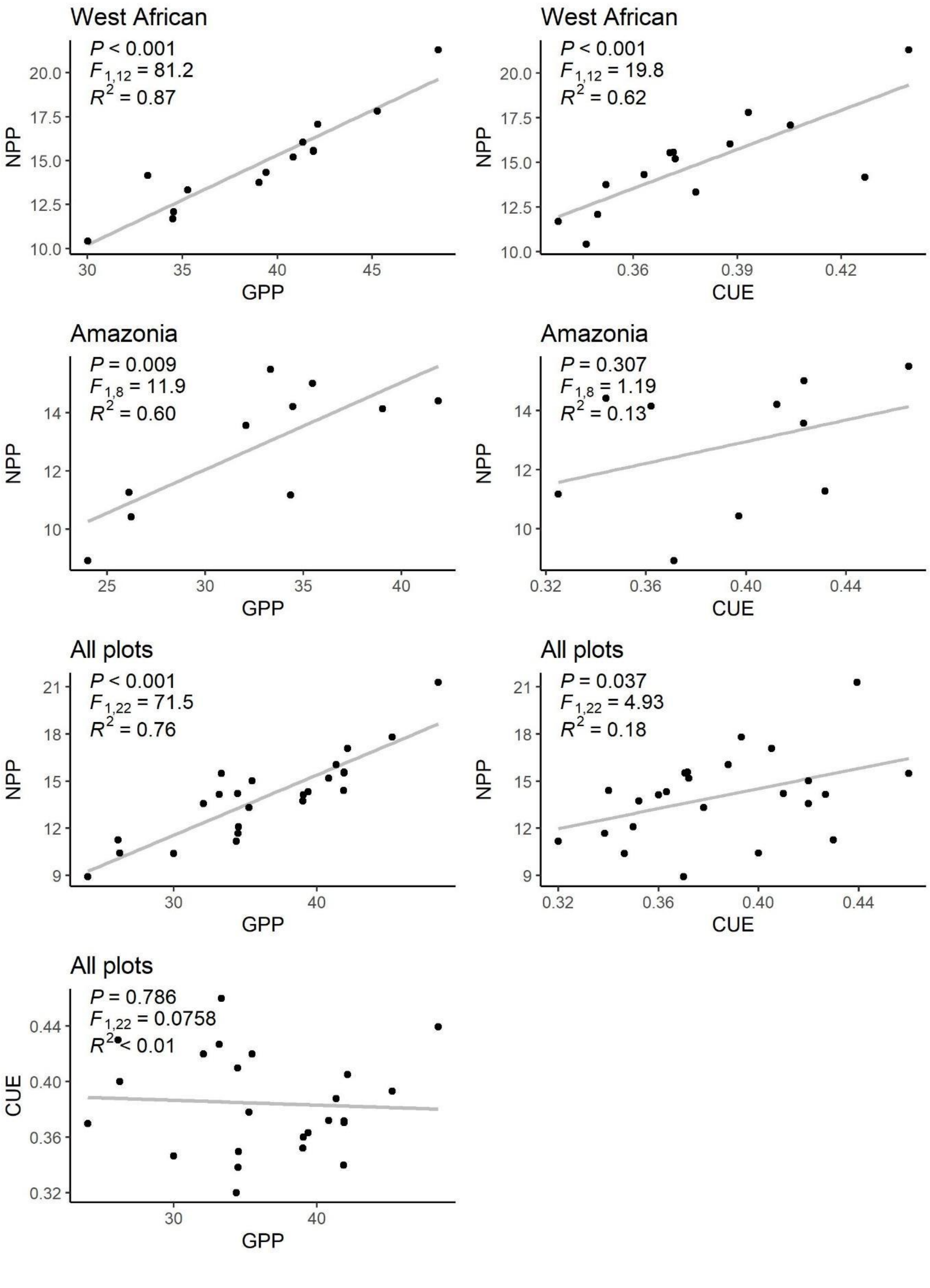

Figure S 1. Linear regression between gross primary productivity (GPP) and net primary productivity (NPP) both in unit (Mg C ha^{-1} yr^{-1}). The figure shows results for West African plots, Amazonian plots and all plots together.

Supplementary 3 Plots information

Table S 1 Study plots information. All are one-hectare plots. The table is also available on online source data deposit.

Plot_code	RH (%)	Rdark shade	Rdark sun	vwc (%)	MCWD (mm)	MAT (°C)	MAP (mm)	Lat	Lon
ALP-01	89.2	-0.67	-0.56	26.8	-6	25.2	2689	-3.95	-73.4333
ALP-30	89.2	-0.79	-0.89	10.8	-6	25.2	2689	-3.9543	-73.4267
ANK-03	91	-1.22	-1.71	11.63	-13	25	2050	5.27102	-2.69234
ANK-02	91	-1.25	-1.75	6.78	-13	25	2050	5.268485	-2.695035
ANK-01	91	-1.18	-1.69	5.96	-13	25	2050	5.267868	-2.693635
CAX-06	82.4	-0.44	-0.44	27.1	-203	25.8	2311	-1.7369	-51.46194
CAX-04	82.4	-0.365	-0.365	22.4	-203	25.8	2311	-1.716	-51.457
TAM-06	89.6	-0.495	-0.53	35.5	-259	24.4	1900	-12.8385	-69.296
TAM-05	89.6	-0.79	-0.655	21.8	-259	24.4	1900	-12.8309	-69.2705
BOB-03	83.9	-1.56	-1.86	11.37	-374	25.7	1500	6.694531	-1.293695
BOB-04	83.9	-1.47	-1.88	9.42	-374	25.7	1500	6.69096	-1.317038
BOB-05	83.9	-1.47	-1.76	8.64	-374	25.7	1500	6.692606	-1.30727
BOB-06	83.9	-1.47	-1.75	8.19	-374	25.7	1500	6.691368	-1.307001
BOB-02	83.9	-1.54	-1.83	7.87	-374	25.7	1500	6.69147	-1.338429
BOB-01	83.9	-1.57	-1.9	6.4	-374	25.7	1500	6.704713	-1.319068
KEN-01	80.8	-0.655	-0.815	19.7	-386	23.4	1310	-16.0158	-62.7301
KEN-02	80.8	no shade	-0.775	16	-386	23.4	1310	-16.0158	-62.7301
KOG-02	79.2	-1.59	-2.08	4.13	-412	26.4	1200	7.262316	-1.149953
KOG-03	79.2	-1.69	-2.12	2.9	-412	26.4	1200	7.306792	-1.156446
KOG-05	79.2	-1.91	-2.39	2.54	-412	26.4	1200	7.305341	-1.164546
KOG-04	79.2	-1.78	-2.32	2.42	-412	26.4	1200	7.302644	-1.180213
KOG-06	79.2	-1.9	-2.34	1.72	-412	26.4	1200	7.329423	-1.15578
TAN-01	74.09	No data	No data	10.8	-482	25	1770	-13.0765	-52.3858
TAN-02	74.09	No data	No data	10.7	-482	25	1770	-13.0765	-52.3858

Note: (1) Leaf dark respiration and relative humidity are from [5,12,22–27] (2) At site CAX, leaf dark respiration is from [28], information on sun/shade leaves were not provided. (3) For CAX, leaf dark respiration is standardized to 25 degrees. For ANK, BOB and KOG, leaf dark respiration is standardized to mean annual air temperature. Information on temperature standardization is not provided in the cited publications for remaining sites. (4) Volumetric water content (vwc) at site ANK, BOB and KOG are measurements of topsoil (12 cm) only, but vwc at site ALP, TAM, KEN, CAX, and TAN are measurements of top 30 cm. This data is provided for reference only and are not suggested for any quantitative analysis. (5) RH = relative humidity; Rdark = leaf dark respiration for shade or sun leaves; vwc=

volumetric water content, indicating surface soil moisture; MCWD = maximum climatological water deficit; MAT= mean annual air temperature; MAP = mean annual air precipitation; Lat = latitude; Lon = longitude. Plots are ranked by MCWD.

Table S1 continued

Plot	Elev	PPFD	Trees	P	N	C	Ca	K	Mg	Sand	Clay
	(m)	(mol/m2/day)	(#/ha)	(mg/kg)	(%)	(%)	(mg/kg)	(mg/kg)	(mg/kg)	(%)	(%)
ALP-01	120	29.49	589	125.6	0.1	1.19				65	15
ALP-30	150	29.49	479	37.6	0.08	1.13				82	2
ANK-03	86	30.97	517	109.7	0.12	1.91	40	33.7	29.2	75.9	12.8
ANK-02	124	30.97	445	146.8	0.17	2.61	26.8	32.3	42	63.1	21.6
ANK-01	114	30.97	476	146.8	0.17	2.61	26.8	32.3	42	63.1	21.6
CAX-06	47	32.33	448	178.5	0.13	1.68				32.54	53.76
CAX-04	47	32.33	no data	37.4	0.06	0.83				83.69	10.68
TAM-06	215	27.22	667	528.8	0.17	1.2				2	46
TAM-05	223	27.22	556	256.3	0.16	1.51				40	44
BOB-03	294	35.58	631								
BOB-04	272	35.58	506								
BOB-05	246	35.58	527								
BOB-06	278	35.58	545								
BOB-02	281	35.58	789	258.3	0.16	1.71	657.6	49	133.7	46.7	28.8
BOB-01	277	35.58	519	77.8	0.09	0.8	306.3	47.6	79.7	64.2	6.7
KEN-01	384	33.46	465	447.1	0.22	2.4				58.05	19.13
KEN-02	384	33.46	470	244.7	0.17	2				55.48	18.25
KOG-02	229	44.26	197	67.2	0.06	0.72	378.9	42.5	75.6	82.4	2.3
KOG-03	198	44.26	216								
KOG-05	221	44.26	193	81.9	0.04	0.62	237.1	28.7	81.3	76.9	4.3
KOG-04	230	44.26	234	74.6	0.05	0.67	308	35.6	78.7	79.7	3.3
KOG-06	195	44.26	202								
TAN-01	385	38.22	370	147	0.16	2.55				45.73	48.9
TAN-02	385	38.22	387	147	0.16	2.55				45.73	48.9

Note: Plots are ranked by MCWD; Empty cells are due to the lack of data. Elev, elevation; MAP, mean annual precipitation; PPFD, photosynthetic photon flux density, calculated from shortwave radiation *0.45; Trees, total number of trees larger than 10cm diameter at breast height, as this number changes from year to year, the first year is picked if a plot has multiple years censuses; Soil nutrients (P, phosphorus; N, nitrogen; C, carbon; Ca, calcium; Mg, magnesium), and soil percentage of sand (Sand) and of clay (Clay). Parts of the data are from [5,21,27,29]

Table S1 continued

Plot	Silt	Seasonality index	Asat	Amax	Leaf lifespan
	(%)	unitless	(μmol m–2 s–1)	(μmol m–2 s–1)	(year)
ALP-01	20	0.23	7.5 ± 4.4	17.4 ± 6.1	
ALP-30	16	0.23	6.7 ± 3.2	16.1 ± 6.2	
ANK-03	11.4	0.33	5.954 ± 0.53	18.57	
ANK-02	15.3	0.33			0.827
ANK-01	15.3	0.33	4.568 ± 0.27	15.88	0.768
CAX-06	13.7	0.68			1.45
CAX-04	5.64	0.68			3
TAM-06	52	0.58	9.4 ± 3.5	22.6 ± 3.6	1.42
TAM-05	17	0.58	9.5 ± 2.7	22.2 ± 3.6	1.3
BOB-03		0.44			
BOB-04		0.44			
BOB-05		0.44			
BOB-06		0.44			
BOB-02	24.5	0.44	7.210 ± 0.24	21.738	0.432
BOB-01	26.8	0.44	7.402 ± 0.49	21.823	0.341
KEN-01	22.82	0.55			1.05
KEN-02	26.27	0.55			1.01
KOG-02	15.34	0.53	6.985 ± 0.26	21.017	0.801
KOG-03		0.53			0.653
KOG-05	18.71	0.53	7.728 ± 0.53	20.654	0.554
KOG-04	17.02	0.53	7.470 ± 0.53	22.866	0.555
KOG-06		0.53			
TAN-01	5.37	0.78			1.04
TAN-02	5.37	0.78			1.04

Note: Asat is light-saturated net photosynthesis measured under 400 ppm atmospheric CO2, Amax is light-saturated net photosynthesis measured under 2000 ppm atmospheric CO2. Measurements source from previous studies[5,30]. Leaf lifespan sources from these[21,31]. Seasonality Index is calculated as the sum of the absolute distance between monthly rainfall and mean rainfall, following[32], using ERA5-Land monthly precipitation [33].

Table S 2 Mean and Standard error (SE) of gross primary production (GPP) and its components across study plots for Amazonia and West African forests. Please note that the standard error is associated with the mean across plots. The standard error thus represents spatial variation, different

to the standard error in Figure 2 which sources from error propagation and represents measurement uncertainty. Welch two sample t-test was used to examine the difference between Amazonian and West African forests. Soil moisture is soil volumetric water content (%) measured from 12cm depth. See Supplementary Data 1 for full names and definitions of carbon budget components. All units are MgC ha^{-1} year^{-1}

	Amazonia Mean	Amazonia SE	West African Mean	West African SE	Welch two sample t-test
GPP	32.71	1.82	39.13	1.35	t = -2.8304, df = 17.884, p-value = 0.01114
CUE	0.39	0.01	0.38	0.01	t = 0.92143, df = 14.792, p-value = 0.3716
NPP	12.86	0.71	14.87	0.74	t = -1.9682, df = 21.592, p-value = 0.06202
NPP_all_stem	3.01	0.21	2.37	0.28	t = 1.8598, df = 21.758, p-value = 0.07649
NPP_fine_litter_fall	5.22	0.31	5.36	0.67	t = -0.17942, df = 18.042, p-value = 0.8596
R_autotrophic	19.84	1.35	24.26	0.71	t = -2.8859, df = 13.952, p-value = 0.01201
NPP_fineroot	3.06	0.35	3.54	0.24	t = -1.119, df = 16.996, p-value = 0.2787
R_rhizosphere	4.24	0.37	2.77	0.25	t = 3.3144, df = 16.812, p-value = 0.00415
R_leaf	6.87	0.70	12.40	0.51	t = -6.3666, df = 17.709, p-value = 5.794e-06
R_stem	7.23	0.64	7.51	0.27	t = -0.40703, df = 12.267, p-value = 0.691
Soil_Moisture	20.16	2.62	6.43	0.88	

Table S 3 Mean and Standard error (SE) of percentage allocation of net primary production (NPP) and autotrophic respiration (R) across study plots for Amazonia and West African forests. Paired t-test was used to examine the difference between Amazonia and West African forests (24 one-hectare plots, so df=23). All values are unitless. Measurements source from previous studies[5,30]. 'Overall' is an average across Amazonia and West Africa.

	Overall SE	Overall Mean	Amazonia SE	West African SE	Amazonia Mean	West African Mean
NPP canopy allocation	0.023	0.421	0.024	0.036	0.412	0.427
NPP woody allocation	0.018	0.311	0.017	0.027	0.333	0.296
NPP fine root allocation	0.015	0.259	0.023	0.020	0.234	0.278
R canopy allocation	0.021	0.441	0.024	0.011	0.345	0.510
R wood allocation	0.015	0.403	0.029	0.012	0.442	0.376
R fine root allocation	0.013	0.155	0.012	0.010	0.213	0.114
Paired T test comparing NPP allocation:<br>(1) canopy to woody: t = 2.8818, df = 23, p-value = 0.00842<br>(2) canopy to fine root: t = 4.7066, df = 23, p-value = 9.672e-05<br>(3) woody to fine root: t = -2.277, df = 23, p-value = 0.03241<br><br>Paired T test comparing Autotrophic allocation:<br>(1) canopy to woody: t = 1.117, df = 23, p-value = 0.2755<br>(2) canopy to fine root: t = 9.405, df = 23, p-value = 2.401e-09<br>(3) woody to fine root: t = -12.929, df = 23, p-value = 4.923e-12						

Table S 4 light-saturated net photosynthesis measured under 400 ppm atmospheric CO2 (Asat), and light-saturated net photosynthesis measured under 2000 ppm atmospheric CO2 (Amax) of common species at study the study sites. Unit is (µmol m^{-2} s^{-1}). Basal Area is the total basal area of a given species (unit mm^2); Count is the number of individuals of a given species. Please note that only some common species are provided. For light saturated assimilation rate at 400 ppm, Asat (umol CO_2 m-2 s-1) and at 2000ppm, Amax (umol CO_2 m-2 s-1), The branch that had been cut was promptly placed in water and recut. To measure leaf gas exchange traits, an open flow gas exchange system (LI-6400XT, Li-Cor Inc., Lincoln, NE, USA) was used. Three leaves were selected from each tree and analyzed for Asat and Amax. The photosynthetic photon flux density was set at 2000 µmol m-2 s-1. The block temperature was kept constant at 30º C throughout the sampling period, which was similar to the ambient air temperature.

Plot	Genus	Species	Asat	BasalArea	Count	Amax

ANK-01	Cynometra	ananta	4.537	6951650	40	18.509
ANK-01	Uapaca	corbisieri	4.713	2995388	14	13.658
ANK-01	Strephonema	pseudocola	4.96	1758334	19	15.805
ANK-01	Heritiera	utilis	5.526	1558052	17	16.909
ANK-01	Dacryodes	klaineana	4.091	1120238	18	13.224
ANK-03	Protomegabaria	macrophylla	4.252	3968181	101	15.327
ANK-03	Uapaca	corbisieri	8.015	3061876	9	18.643
ANK-03	Strephonema	pseudocola	5.286	2154328	18	17.068
ANK-03	Heritiera	utilis	7.949	1575264	19	23.802
ANK-03	Cleistopholis	patens	6.961	1525673	9	24.293
BOB-01	Celtis	mildbraedii	6.828	5161832	76	21.007
BOB-01	Nesogordonia	papaverifera	8.709	2336989	26	22.73
BOB-01	Triplochiton	scleroxylon	8.572	1959843	11	25.227
BOB-01	Hannoa	klaineana	5.903	1566631	20	22.46
BOB-01	Cola	gigantea	5.931	720700.2	16	14.306
BOB-02	Triplochiton	scleroxylon	8.385	6031773	25	24.849
BOB-02	Celtis	mildbraedii	4.609	4804028	82	16.548
BOB-02	Funtumia	elastica	10.006	2945646	153	27.193
BOB-02	Celtis	zenkeri	4.431	1828924	55	16.593
BOB-02	Nesogordonia	papaverifera	7.229	1782720	38	20.36
KOG-02	Dacryodes	klaineana	4.495	3277066	18	15.695
KOG-02	Cola	gigantea	6.684	2875349	23	19.891
KOG-02	Ceiba	pentandra	8.519	1802779	9	24.131
KOG-02	Afzelia	africana	7.505	1610019	7	22.846
KOG-02	Khaya	grandifoliola	5.766	1132927	4	20.101
KOG-04	Pterocarpus	erinaceus	7.688	1667214	25	22.3
KOG-04	Manilkara	multinervis	8.244	1629990	14	21.949
KOG-04	Sterculia	tragacantha	7.272	1610797	63	23.24
KOG-04	Lannea	velutina	3.458	981867.9	8	14.945
KOG-04	Afzelia	africana	12.393	952962.9	3	32.673
KOG-05	Pterocarpus	erinaceus	8.394	2578922	24	22.796
KOG-05	Anogeissus	leiocarpus	10.974	2066544	11	22.033
KOG-05	Terminalia	schimperiana	7.785	1563494	15	21.787
KOG-05	Vitellaria	paradoxa	4.387019	1344279	10	15.46
KOG-05	Bridelia	ferruginea	5.380399	918256.1	51	20.02857
TAM-05	Ampelocera	edentula	6			17.2
TAM-05	Bixa	arborea	13			22.6
TAM-05	Ocotea	bofo	9.5			20.6
TAM-05	unidentified	unidentified	6.6			21.2

TAM-05	Pouteria	torta subsp. tuberculata	6.8			25.9
TAM-05	Huberodendron	switenioides	10.6			20.5
TAM-05	Miconia	pyrifolia	11.9			28.7
TAM-05	Sloanea	brevipes	11.5			20.7
TAM-06	Sapium	marmieri	7.6			28
TAM-06	Inga	alba	7.3			22
TAM-06	Ficus	schultesii	13.2			23
TAM-06	Pterocarpus	rohrii	7.1			24.8
TAM-06	Pseudolmedia	laevis	7.4			19.7
TAM-06	unidentified	unidentified	7.2			24.4
TAM-06	Sorocea	pileata	9.1			22.7
TAM-06	Dipteryx	alata	16.4			26.4
TAM-06	Sorocea	trophoides	9.9			20.4
TAM-06	Bertolletia	excelsa	14.8			.
TAM-06	Brosimum	sp.	4			14
TAM-06	Celtis	schippii	9.8			23
TAM-06	Clarisia	racemosa	8.2			22.4
ALP-30	Tachigali	bracteosa	4.4			22.9
ALP-30	Brosimum	potabile	5.6			16.5
ALP-30	Sloanea	floribunda	5.6			13.6
ALP-30	Micrandra	spruceana	2			7.1
ALP-30	Simarouba	amara	8.4			20.5
ALP-30	Humiria	balsamifera	7.6			15.7
ALP-30	Ocotea	aciphylla	8.2			16.2
ALP-30	Aspidosperma	desmanthum	10			27.4
ALP-30	Diplotropis	sp	13.6			31
ALP-30	Guatteria	decurrens	5.7			14.7
ALP-30	Micrandra	elata	2.5			11
ALP-30	Ocotea	myriantha	4.6			14.3
ALP-30	Aspidosperma	excelsum	3.9			21.4
ALP-30	Calyptranthes	bipennis	3.9			12.8
ALP-30	Aniba	perutilis	8.2			15.3
ALP-30	Macrolobium	microcalyx	7.7			8.5
ALP-30	Virola	pavonis	12.7			16.6
ALP-30	Licania	unguiculata	11.1			18.5
ALP-30	Tapirira	guianensis	6.5			12.2
ALP-30	Roucheria	schomburgkii	6.1			15.6
ALP-30	Emmotum	floribundum	2.9			5.6
ALP-01	Dipteryx	micrantha	11.4			16.6

ALP-01	Pouteria	subrotata	11.6			26.7
ALP-01	Licania	arachnoidea	6.9			7.5
ALP-01	Guatteria	schomburgkian a	2.9			22.1
ALP-01	Minquartia	guianensis	9.7			19.3
ALP-01	Iryanthera	lancifolia	12.7			21.9
ALP-01	Hevea	pauciflora	0.9			4.5
ALP-01	Chaunochiton	kappleri	7.5			17.7
ALP-01	Cespedesia	spathulata	4.2			22.5
ALP-01	Taralea	oppositifolia	1.9			7
ALP-01	Brosimum	rubescens	2.9			12
ALP-01	Swartzia	polyphylla	7.4			17.9
ALP-01	Ruptiliocarpon	caracolito	5.5			15.6
ALP-01	Caraipa	punctulata	9.5			23.1
ALP-01	Senefeldera	inclinata	2.3			18.6
ALP-01	Pourouma	guianensis subsp. guianensi	15.9			19.3
ALP-01	Hevea	pauciflora	10.2			19
ALP-01	Inga	striata	11.9			21.6

Supplementary 4 Carbon budget quantification for West African carbon fluxes

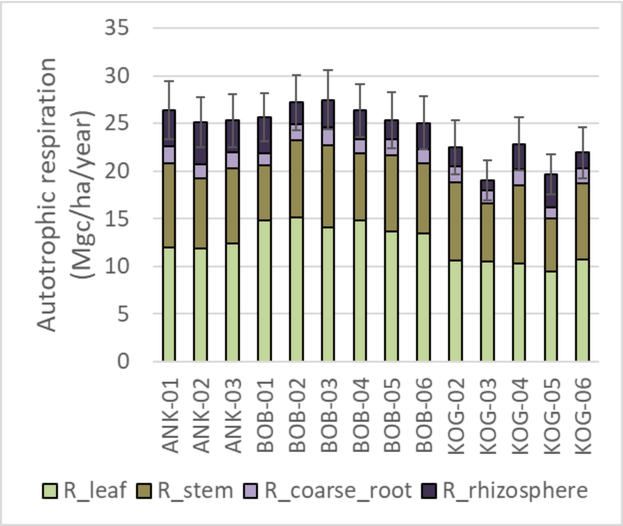

(a)

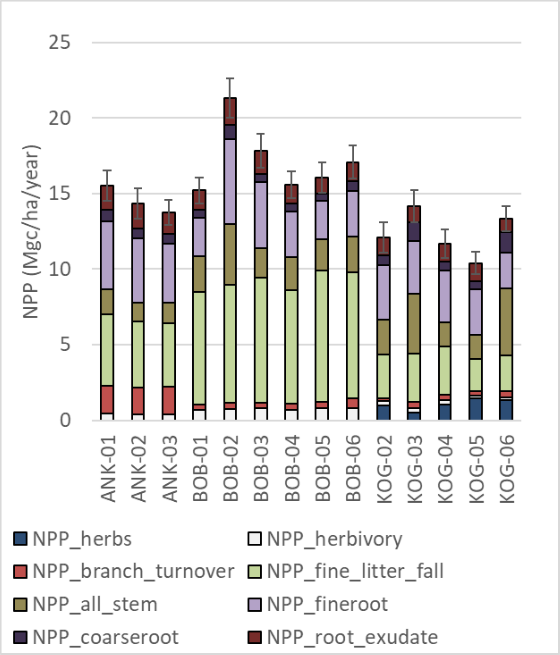

(b)

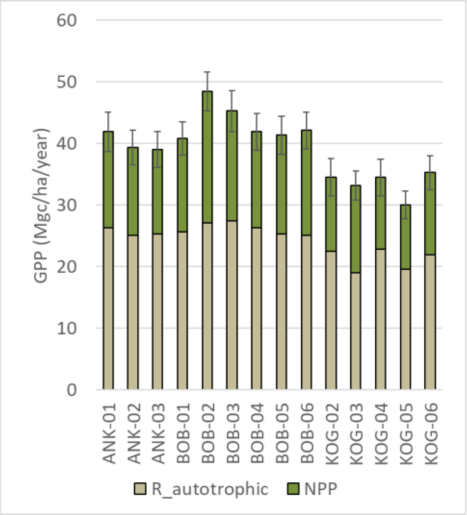

(c)

Figure S2. Components of the carbon budgets. Panel (a) shows autotrophic respiration (R_autotrophic). Panel (b) shows components of net primary production (NPP). Panel (c) shows gross primary production (NPP).

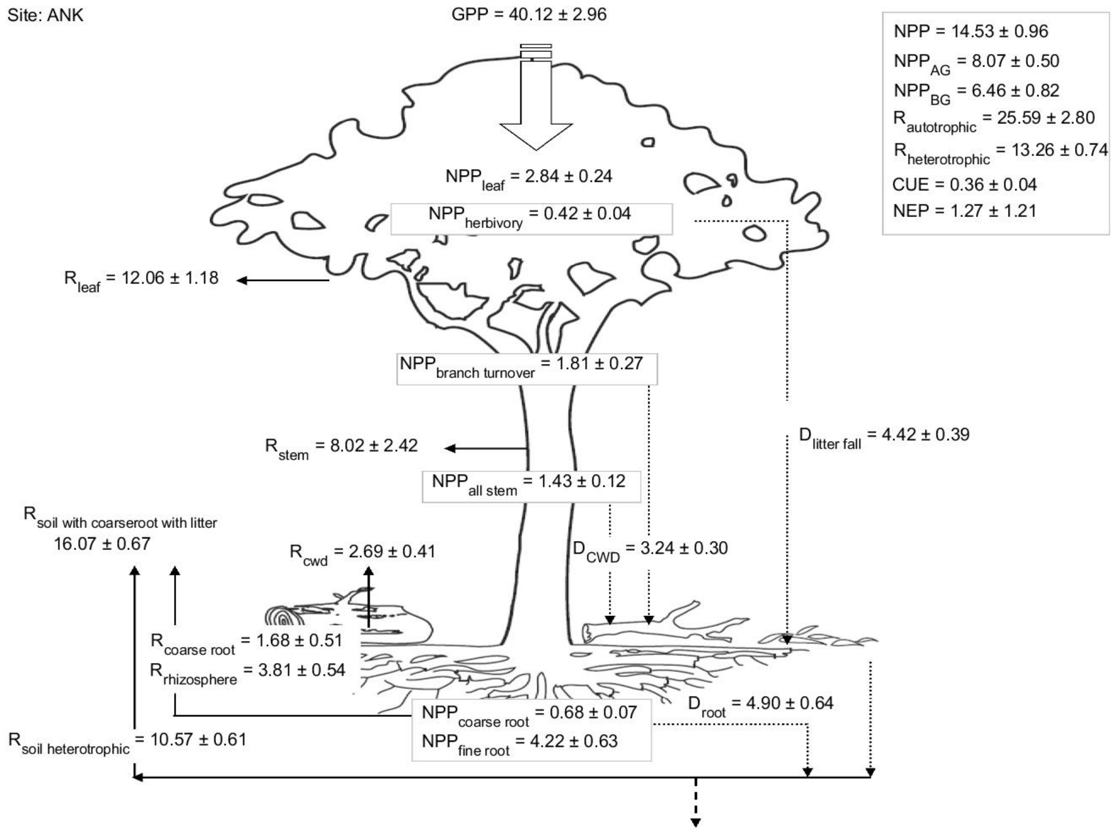
Site: ANK
GPP = 40.12 ± 2.96
NPP = 14.53 ± 0.96
NPP_AG = 8.07 ± 0.50
NPP_BG = 6.46 ± 0.82
R_autotrophic = 25.59 ± 2.80
R_heterotrophic = 13.26 ± 0.74
CUE = 0.36 ± 0.04
NEP = 1.27 ± 1.21
NPP_leaf = 2.84 ± 0.24
NPP_herbivory = 0.42 ± 0.04
R_leaf = 12.06 ± 1.18
NPP_branch turnover = 1.81 ± 0.27
R_stem = 8.02 ± 2.42
D_litter fall = 4.42 ± 0.39
NPP_all stem = 1.43 ± 0.12
R_soil with coarseroot with litter
16.07 ± 0.67
R_cwd = 2.69 ± 0.41
D_CWD = 3.24 ± 0.30
R_coarse root = 1.68 ± 0.51
R_rhizosphere = 3.81 ± 0.54
NPP_coarse root = 0.68 ± 0.07
NPP_fine root = 4.22 ± 0.63
D_root = 4.90 ± 0.64
R_soil heterotrophic = 10.57 ± 0.61

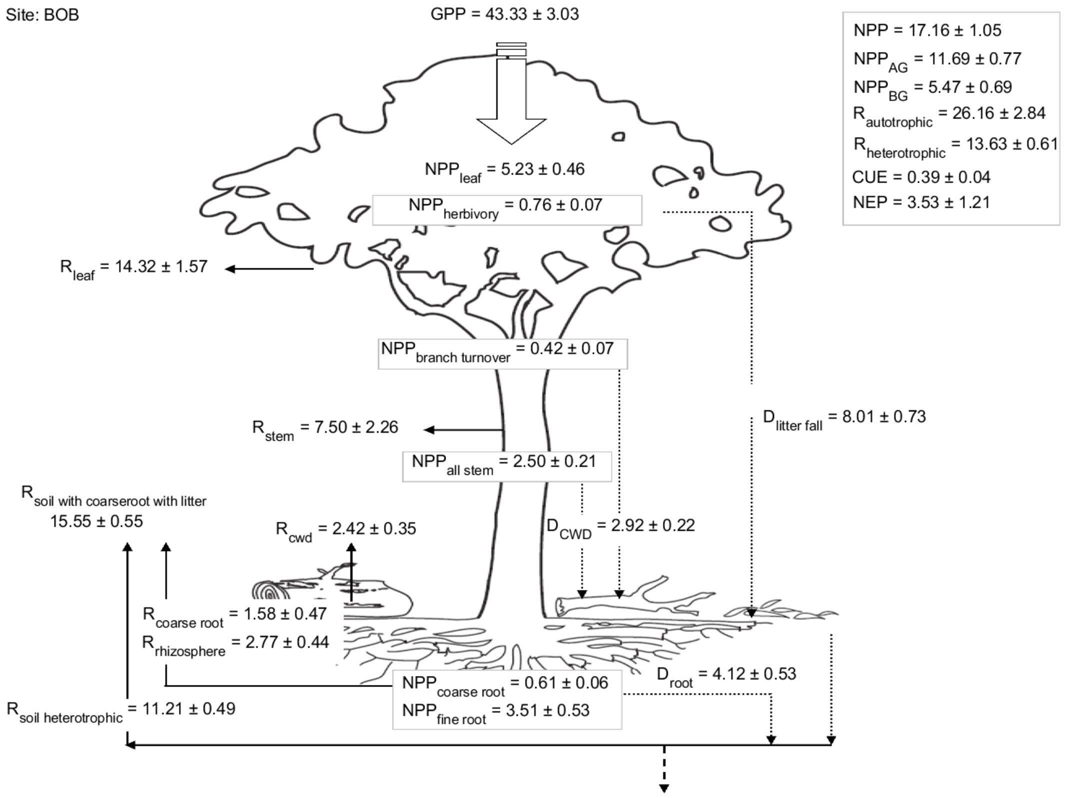
Site: BOB
GPP = 43.33 ± 3.03
NPP = 17.16 ± 1.05
NPP_AG = 11.69 ± 0.77
NPP_BG = 5.47 ± 0.69
R_autotrophic = 26.16 ± 2.84
R_heterotrophic = 13.63 ± 0.61
CUE = 0.39 ± 0.04
NEP = 3.53 ± 1.21
NPP_leaf = 5.23 ± 0.46
NPP_herbivory = 0.76 ± 0.07
R_leaf = 14.32 ± 1.57
NPP_branch turnover = 0.42 ± 0.07
D_litter fall = 8.01 ± 0.73
R_stem = 7.50 ± 2.26
NPP_all stem = 2.50 ± 0.21
R_soil with coarseroot with litter
15.55 ± 0.55
R_cwd = 2.42 ± 0.35
D_CWD = 2.92 ± 0.22
R_coarse root = 1.58 ± 0.47
R_rhizosphere = 2.77 ± 0.44
NPP_coarse root = 0.61 ± 0.06
NPP_fine root = 3.51 ± 0.53
D_root = 4.12 ± 0.53
R_soil heterotrophic = 11.21 ± 0.49

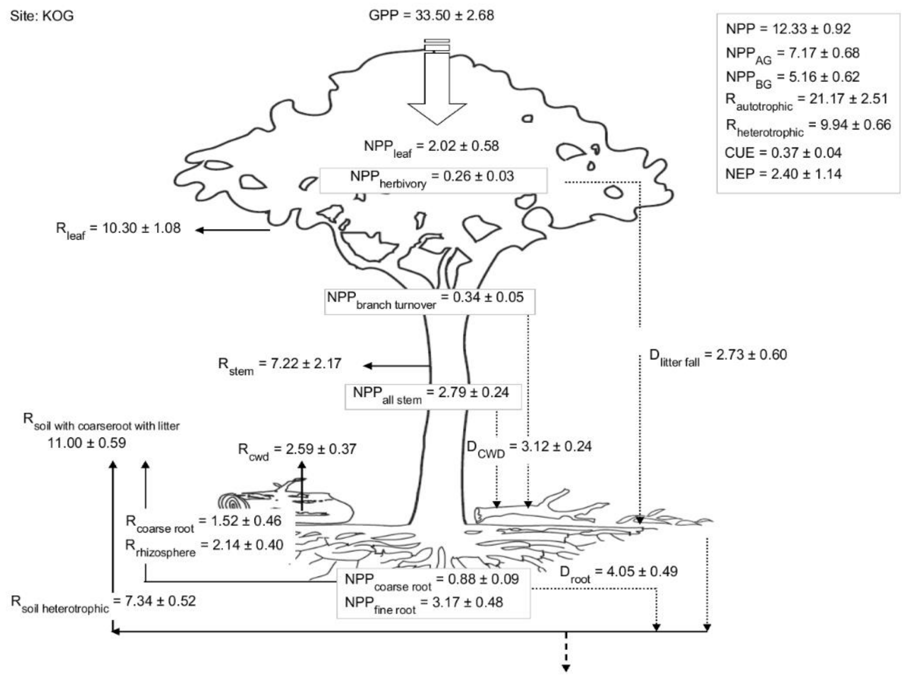

Figure S3. Full carbon budgets visualised on a tree diagram. The diagrams show the magnitude and pattern of key carbon fluxes for ANK (mean of 3 plots) BOB (mean of 6 plots) and KOG (mean of 5 plots)

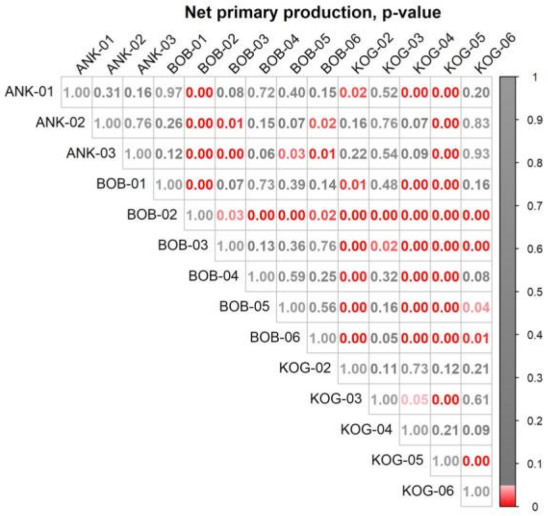

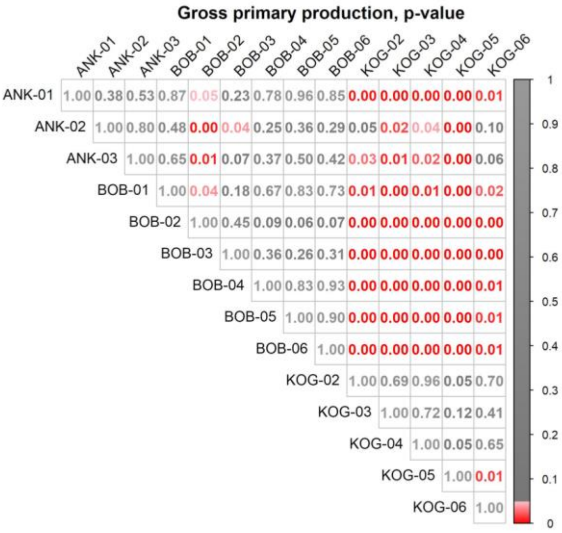

Figure S 4. Z-test and P-value for plot to plot comparison. Z-test was used to compare the difference between plots for net primary production (NPP) and gross primary production (GPP). P values between each plot were shown to illustrate significant (red, at 0.05 threshold) and insignificant (grey).

Supplementary 5 Photographs of the site

Ankasa

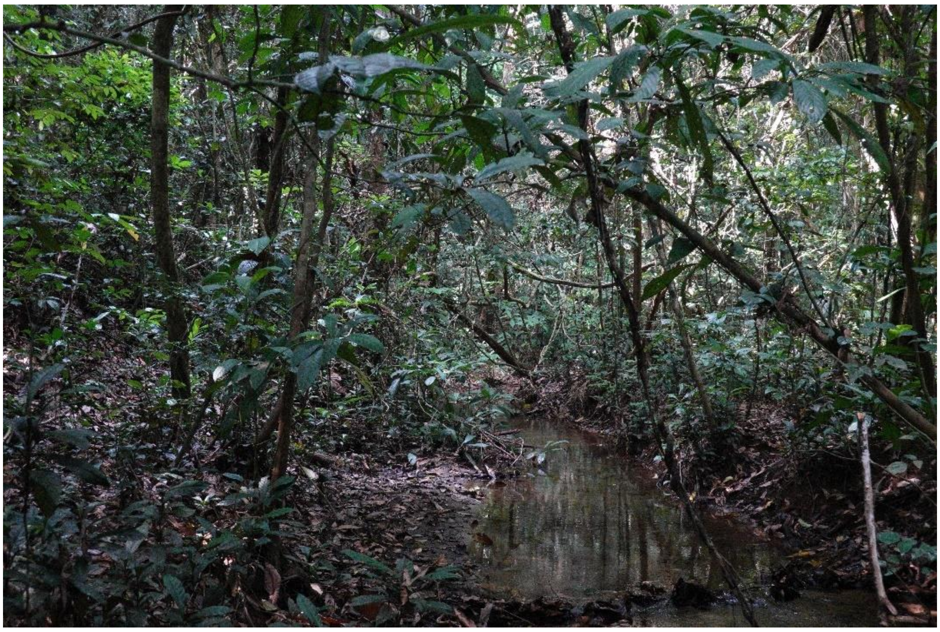

There is a stream running through ANK03 which largely floods the plot in the wet season.

Ankasa - ANK01

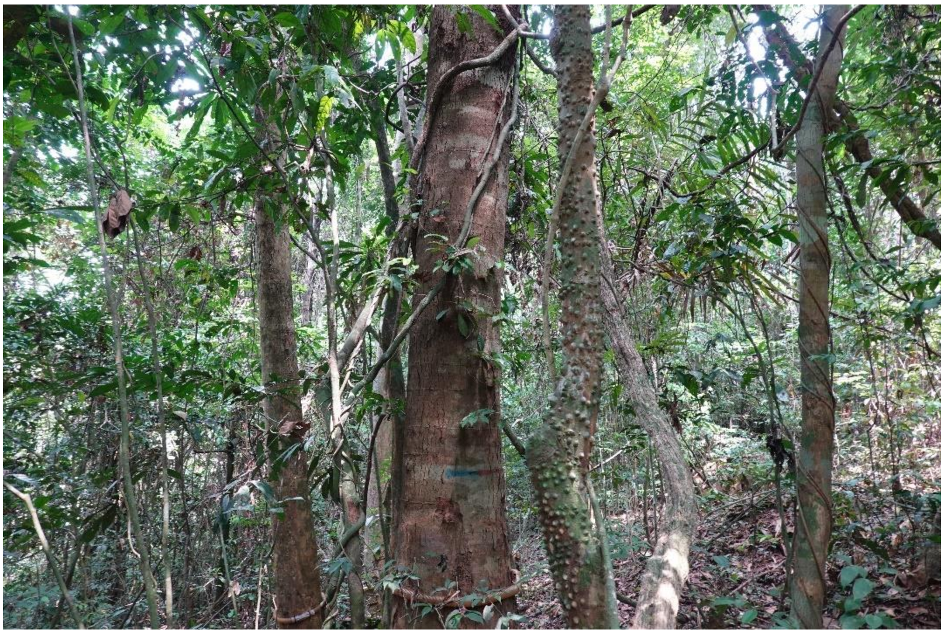

ANK01 and ANK02 are located on well-drained local hilltops.

Bobiri - BOB01

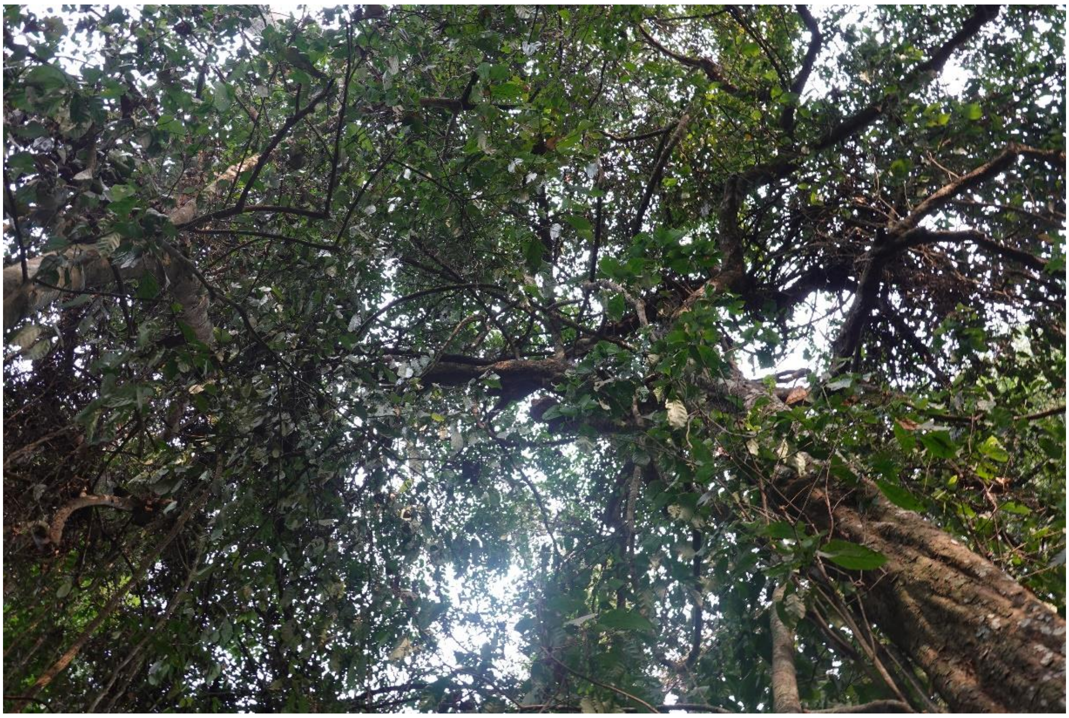

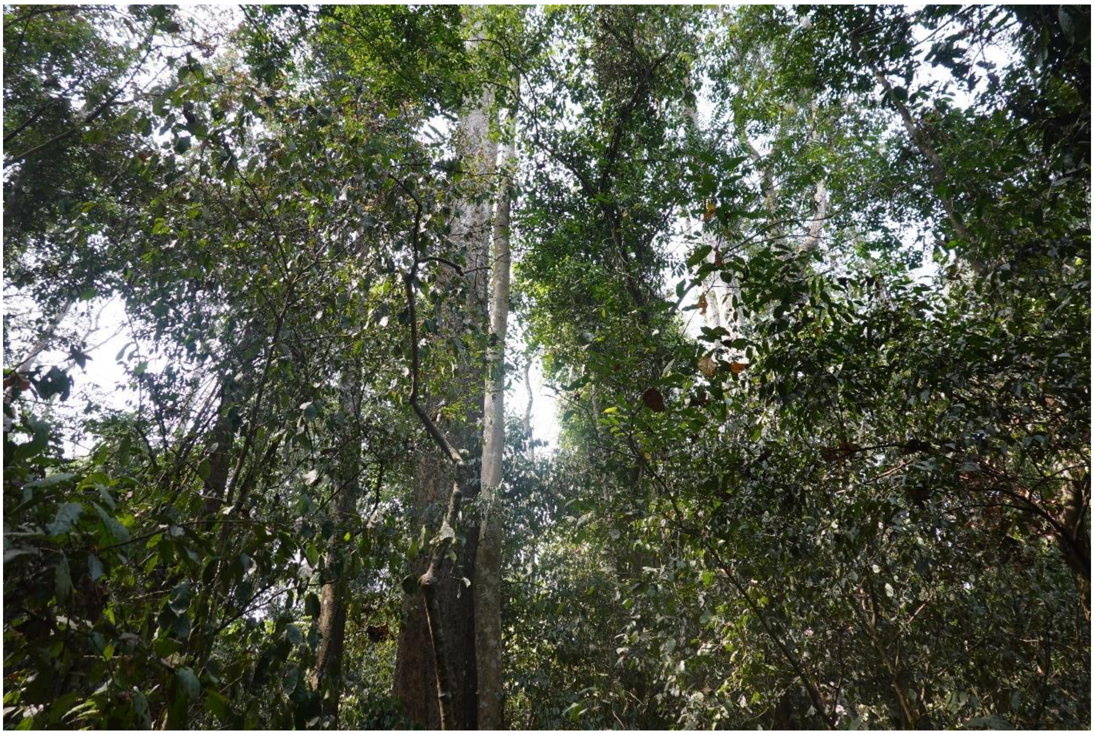

Bobiri - BOB02

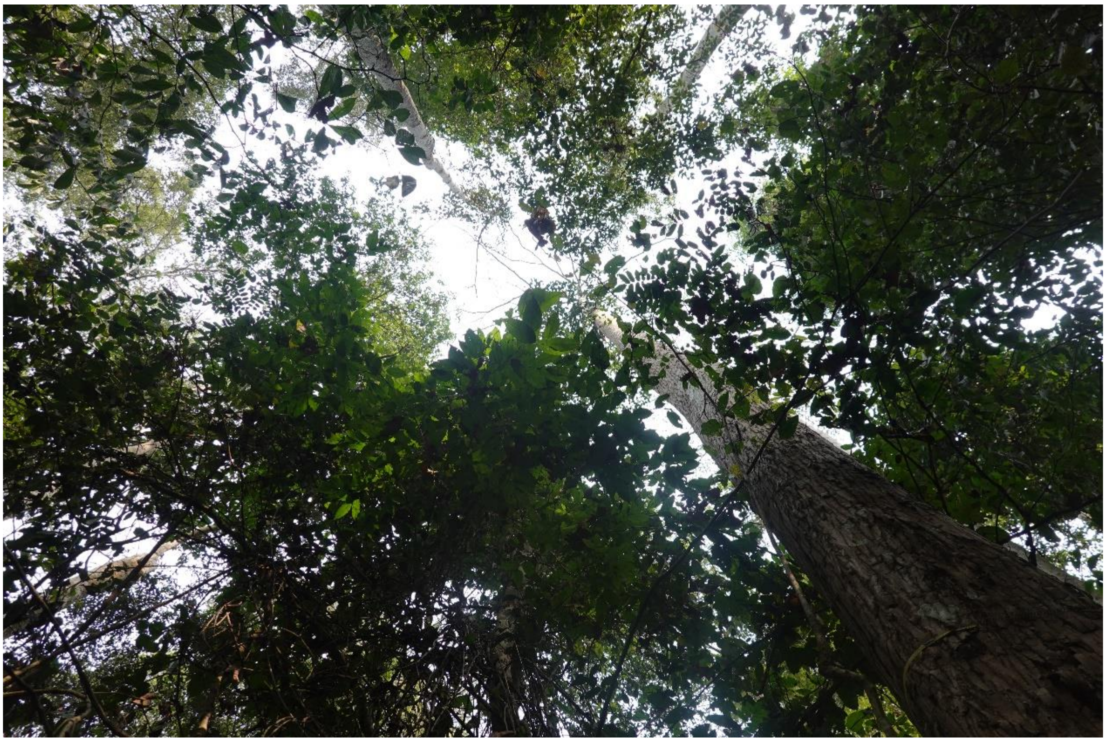

Photo Credit: all photos above were taken by Huanyuan Zhang-Zheng in January 2022.

Kogaye - KOG01

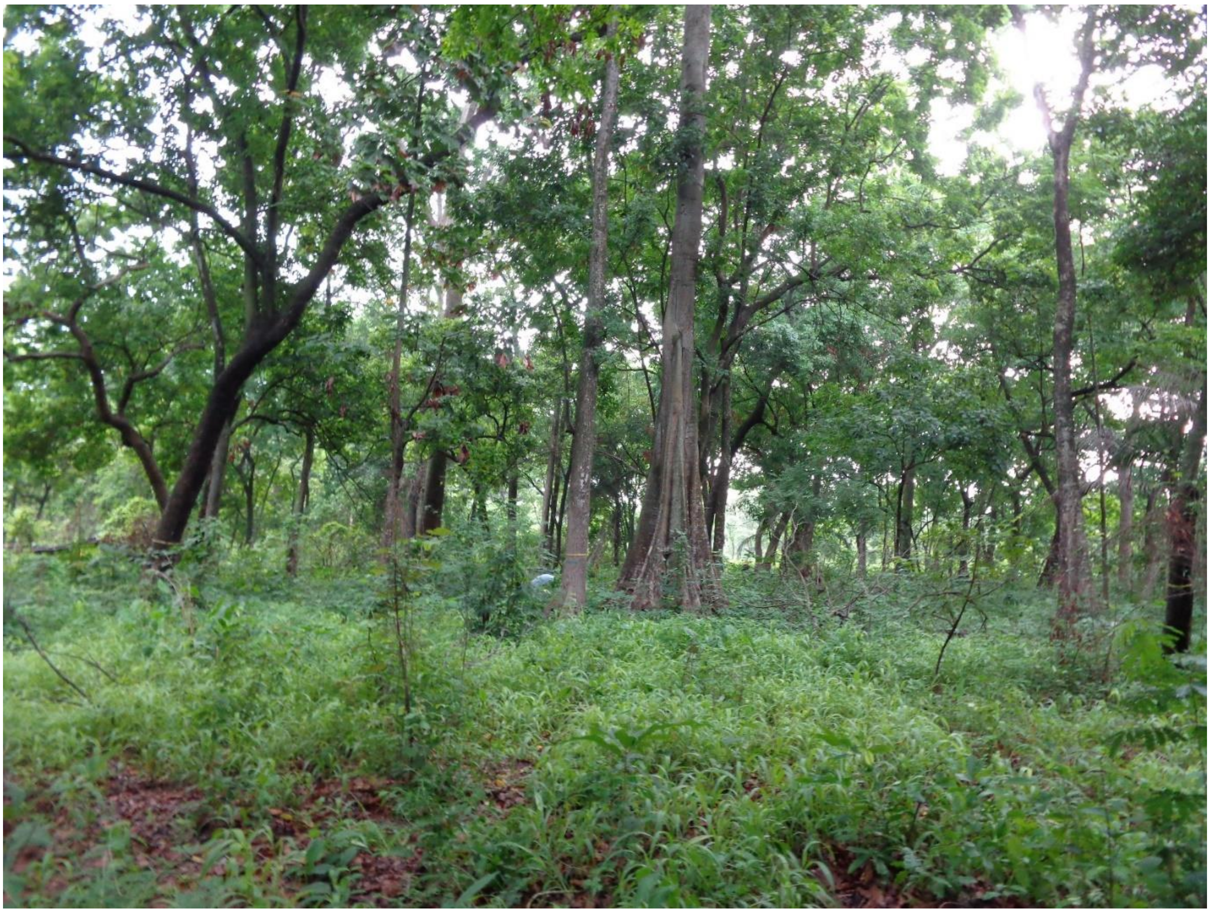

Photo Credit: the photo was shared by Akwasi Duah-Gyamfi. The photo was taken on 16 July 2013,

Kogaye - KOG02

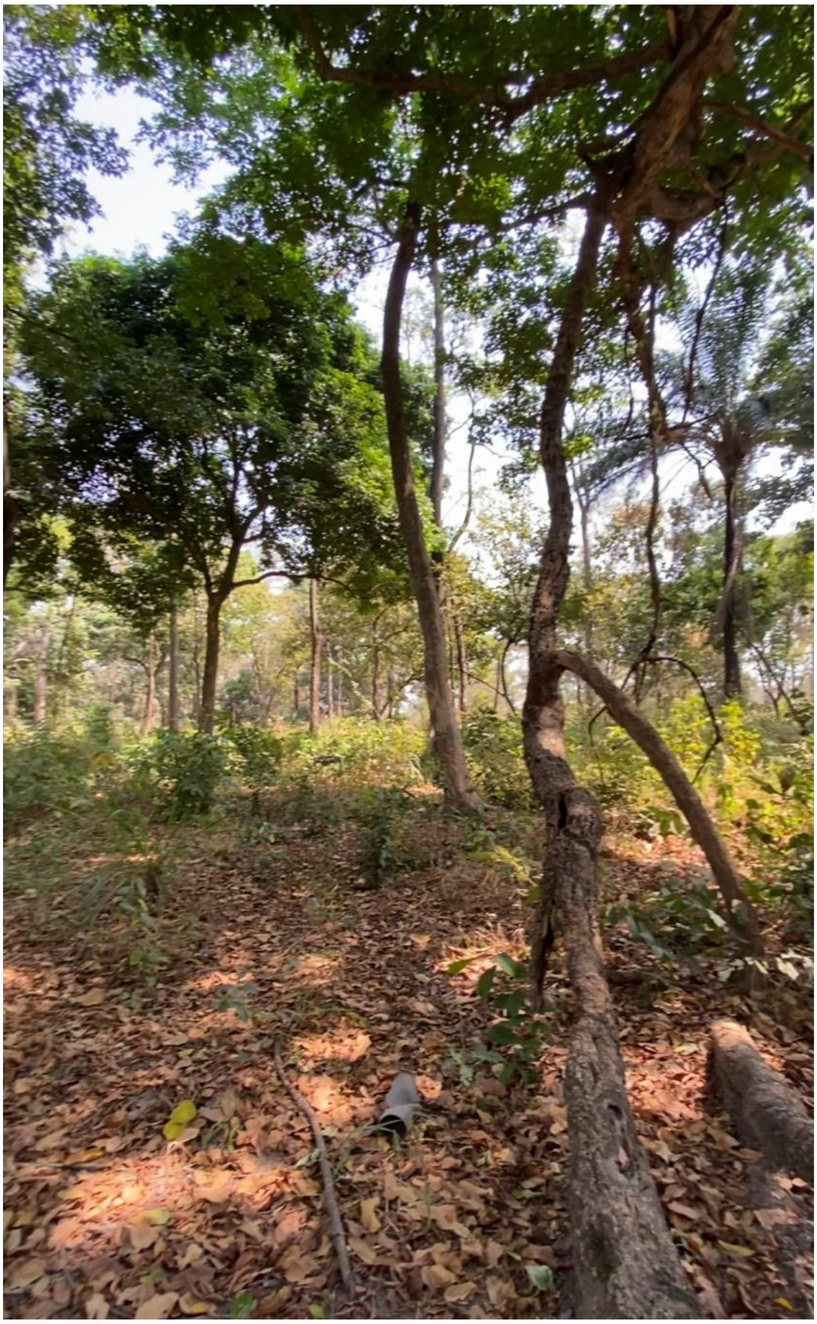

Photo Credit: taken by Huanyuan Zhang-Zheng in January 2022.

Kogaye - KOG04

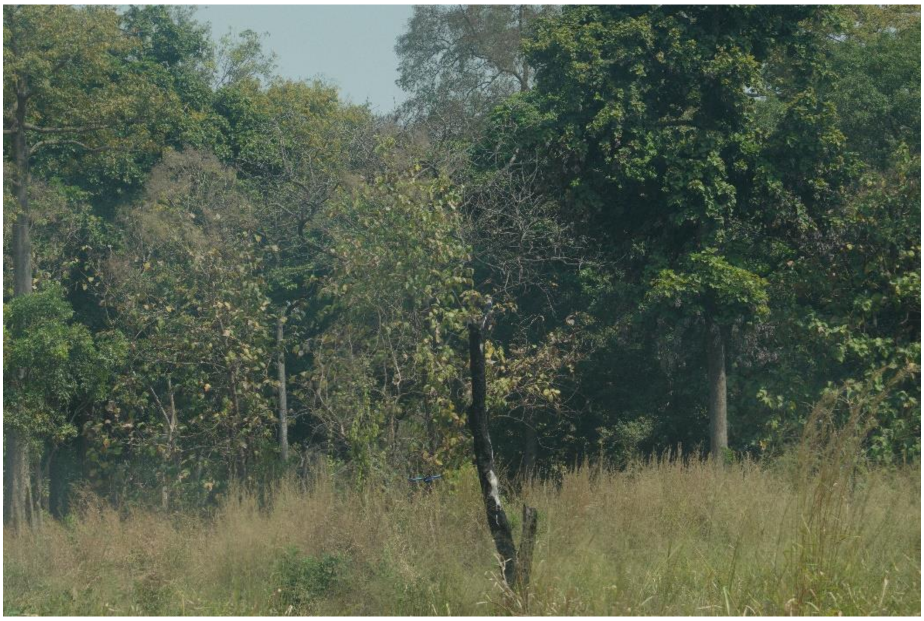

This is at the forest-savanna transition. Photo Credit: taken by Huanyuan Zhang-Zheng in January 2022.

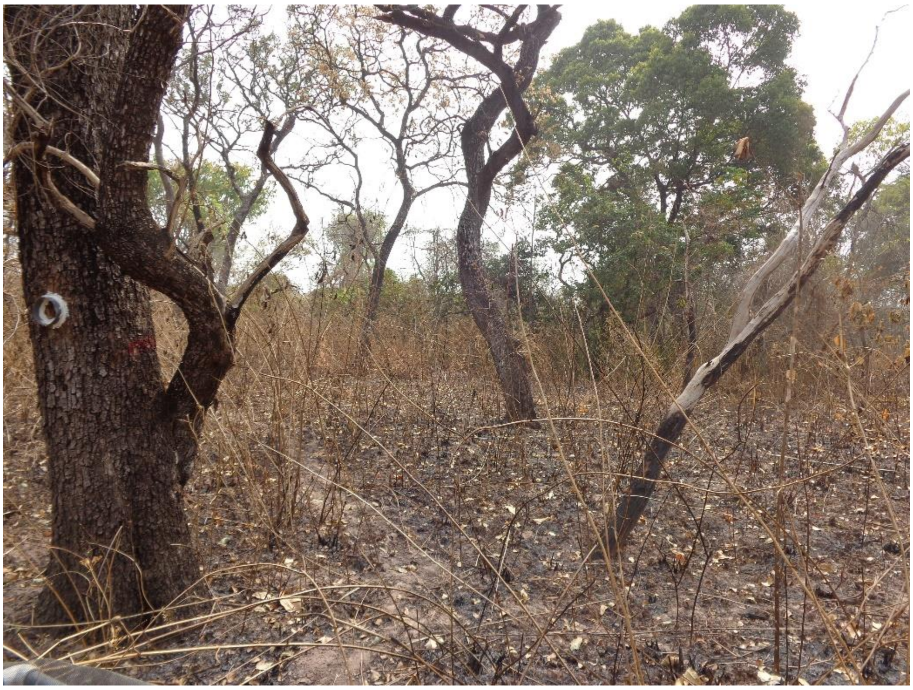

This plot rarely burns (as told by locals), but it looks like this when it does burn. Photo Credit: the photo was shared by Akwasi Duah-Gyamfi. The photo was taken on 03 February 2014,

Kogaye - KOG05

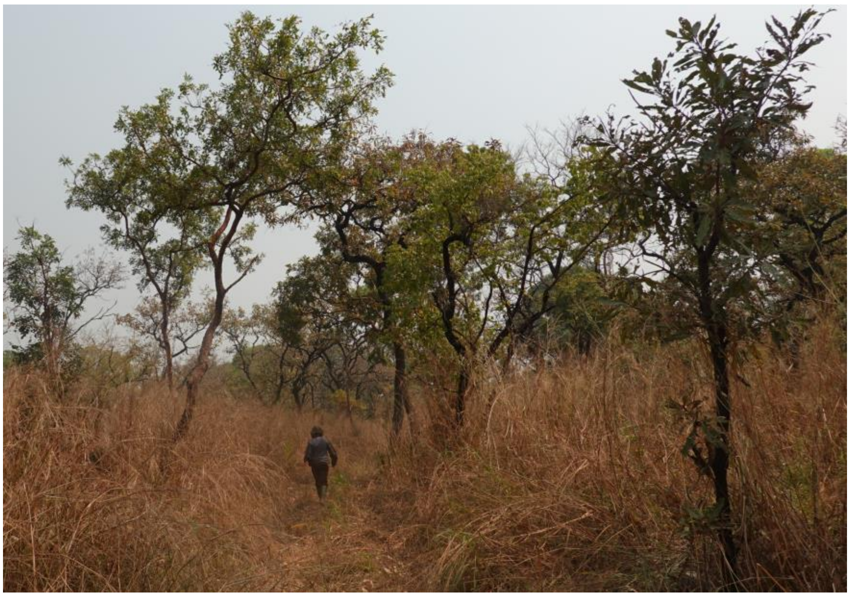

Photo Credit: taken by Huanyuan Zhang-Zheng in January 2022.

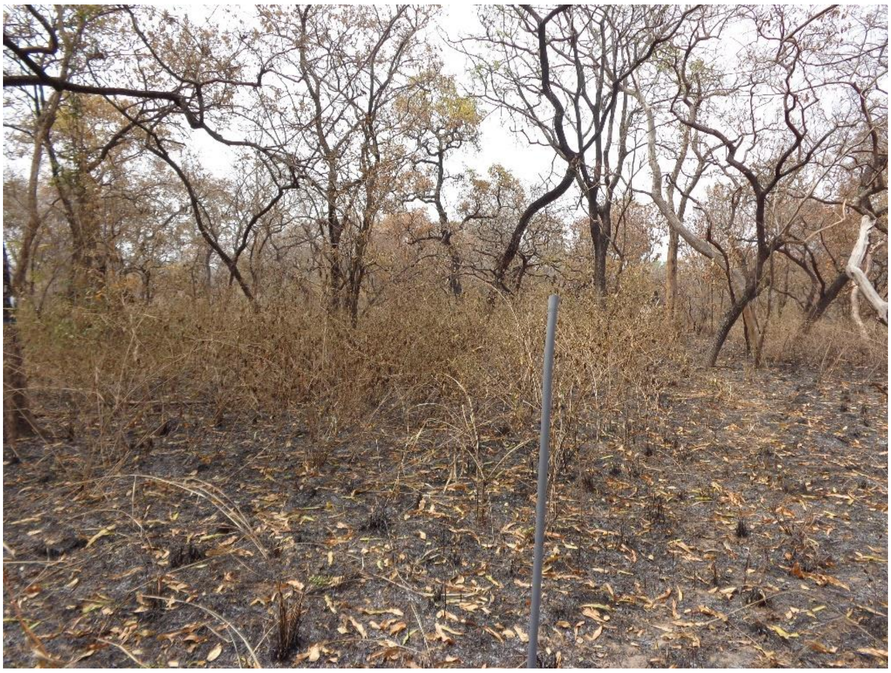

This plot frequently burns. Photo Credit: the photo was shared by Akwasi Duah-Gyamfi. The photo was taken on 06 February 2014,

Supplementary References

1. Berg, S. *et al.* ilastik: interactive machine learning for (bio)image analysis. *Nat Methods* **16**, (2019).
2. Demarez, V., Duthoit, S., Baret, F., Weiss, M. & Dedieu, G. Estimation of leaf area and clumping indexes of crops with hemispherical photographs. *Agric For Meteorol* **148**, (2008).
3. Zhang, Y., Chen, J. M. & Miller, J. R. Determining digital hemispherical photograph exposure for leaf area index estimation. in *Agricultural and Forest Meteorology* vol. 133 (2005).
4. Malhi, Y. *et al.* The Global Ecosystems Monitoring network: Monitoring ecosystem productivity and carbon cycling across the tropics. *Biol Conserv* **253**, 108889 (2021).
5. Zhang-Zheng, H. *et al.* Photosynthetic and water transport strategies of plants along a tropical forest aridity gradient: a test of optimality theory. *bioRxiv* 2023.01.10.523419 (2023) doi:10.1101/2023.01.10.523419.
6. Mujawamariya, M. *et al.* Complete or overcompensatory thermal acclimation of leaf dark respiration in African tropical trees. *New Phytologist* **229**, (2021).
7. Doughty, C. E. & Goulden, M. L. Seasonal patterns of tropical forest leaf area index and CO2 exchange. *J Geophys Res Biogeosci* **114**, (2009).
8. Malhi, Y. *et al.* Comprehensive assessment of carbon productivity, allocation and storage in three Amazonian forests. *Glob Chang Biol* **15**, 1255–1274 (2009).
9. Chambers, J. Q. *et al.* Respiration from a tropical forest ecosystem: Partitioning of sources and low carbon use efficiency. *Ecological Applications* **14**, (2004).

10. Jackson, R. B. *et al.* A global analysis of root distributions for terrestrial biomes. *Oecologia* **108**, (1996).

11. Cairns, M. A., Brown, S., Helmer, E. H. & Baumgardner, G. A. Root biomass allocation in the world's upland forests. *Oecologia* **111**, (1997).

12. Malhi, Y. *et al.* The productivity, metabolism and carbon cycle of two lowland tropical forest plots in south-western Amazonia, Peru. *Plant Ecol Divers* **7**, 85–105 (2014).

13. Metcalfe, D. B. *et al.* Factors controlling spatio-temporal variation in carbon dioxide efflux from surface litter, roots, and soil organic matter at four rain forest sites in the eastern Amazon. *J Geophys Res Biogeosci* **112**, (2007).

14. Riutta, T. *et al.* Major and persistent shifts in below-ground carbon dynamics and soil respiration following logging in tropical forests. *Glob Chang Biol* **27**, 2225–2240 (2021).

15. Finzi, A. C. *et al.* Rhizosphere processes are quantitatively important components of terrestrial carbon and nutrient cycles. *Glob Chang Biol* **21**, (2015).

16. Abramoff, R. Z. & Finzi, A. C. Seasonality and partitioning of Root allocation to rhizosphere soils in a midlatitude forest. *Ecosphere* **7**, (2016).

17. Sun, L. *et al.* Root exudation as a major competitive fine-root functional trait of 18 coexisting species in a subtropical forest. *New Phytologist* **229**, (2021).

18. Chambers, J. Q., Schimel, J. P. & Nobre, A. D. Respiration from coarse wood litter in central Amazon forests. *Biogeochemistry* **52**, (2001).

19. Yoon, T. K., Noh, N. J., Kim, S., Han, S. & Son, Y. Coarse woody debris respiration of Japanese red pine forests in Korea: controlling factors and contribution to the ecosystem carbon cycle. *Ecol Res* **30**, (2015).

20. Mills, M. B. *et al.* Tropical forests post-logging are a persistent net carbon source to the atmosphere. *Proc Natl Acad Sci U S A* **120**, (2023).

21. Moore, S. *et al.* Forest biomass, productivity and carbon cycling along a rainfall gradient in West Africa. *Glob Chang Biol* **24**, e496–e510 (2018).

22. Rocha, W. *et al.* Ecosystem productivity and carbon cycling in intact and annually burnt forest at the dry southern limit of the Amazon rainforest (Mato Grosso, Brazil). *Plant Ecol Divers* **7**, (2014).

23. Araujo-Murakami, A. *et al.* The productivity, allocation and cycling of carbon in forests at the dry margin of the Amazon forest in Bolivia. *Plant Ecol Divers* **7**, 55–69 (2014).

24. del Aguila-Pasquel, J. *et al.* The seasonal cycle of productivity, metabolism and carbon dynamics in a wet aseasonal forest in north-west Amazonia (Iquitos, Peru). *Plant Ecol Divers* **7**, 71–83 (2014).

25. da Costa, A. C. L. *et al.* Ecosystem respiration and net primary productivity after 8-10 years of experimental through-fall reduction in an eastern Amazon forest. *Plant Ecol Divers* **7**, (2014).

26. Doughty, C. E. *et al.* The production, allocation and cycling of carbon in a forest on fertile terra preta soil in eastern Amazonia compared with a forest on adjacent infertile soil. *Plant Ecol Divers* **7**, 41–53 (2014).

27. Oliveras, I. *et al.* The influence of taxonomy and environment on leaf trait variation along tropical abiotic gradients. *Frontiers in Forests and Global Change* **3**, 18 (2020).

28. Metcalfe, D. B. *et al.* Impacts of experimentally imposed drought on leaf respiration and morphology in an Amazon rain forest. *Funct Ecol* **24**, (2010).

29. Malhi, Y. *et al.* The linkages between photosynthesis, productivity, growth and biomass in lowland Amazonian forests. *Glob Chang Biol* **21**, (2015).

30. Bahar, N. H. A. *et al.* Leaf-level photosynthetic capacity in lowland Amazonian and high-elevation Andean tropical moist forests of Peru. *New Phytologist* **214**, 1002–1018 (2017).

31. Flack-Prain, S., Meir, P., Malhi, Y., Smallman, T. L. & Williams, M. Leaf Area Index Changes Explain GPP Variation across an Amazon Drought Stress Gradient. *Biogeosciences Discussions* (2019).

32. Imteaz, M. A. & Hossain, I. Climate Change Impacts on 'Seasonality Index' and its Potential Implications on Rainwater Savings. *Water Resources Management* **37**, (2023).

33. Muñoz-Sabater, J. *et al.* ERA5-Land: A state-of-the-art global reanalysis dataset for land applications. *Earth Syst Sci Data* **13**, (2021).
