# Supplementary material for: Contrasting carbon cycle along tropical forest aridity gradients in West Africa and Amazonia
Source: Nat Commun. 2024 Apr 11;15:3158. doi: 10.1038/s41467-024-47202-x (PMC11009382; doi:10.1038/s41467-024-47202-x)
Supplement: Supplementary file 3 — Description of Additional Supplementary Files [file 41467_2024_47202_MOESM3_ESM.pdf]

Description of Additional Supplementary Files

File Name: Supplementary Data 1
Description: Abbreviation for components of NPP, respiration and detritus carbon flux. See Supplementary Information for detailed field protocol and calculation procedures. Data at site ANK BOB and KOG were retrieved from a previous study 10 except NPP_root_exudate.
